# Salicylaldehyde-Grafted g‑C_3_N_4_ for Enhanced Photocatalytic Degradation of Diclofenac and Antibiotics

**DOI:** 10.1021/acsomega.6c06050

**Published:** 2026-09-25

**Authors:** Yu-Shan Lin, Yu-Shen Lin, I-Cheng Liu, Tai-Chia Chiu, Cho-Chun Hu

**Affiliations:** Department of Applied Science, 63285 National Taitung University, No. 369, Sec. 2, University Road, Taitung City, Taitung County 95092, Taiwan (R.O.C.)

## Abstract

In this study, salicylaldehyde-grafted modified graphitic carbon nitride (modified g-C_3_N_4_, denoted CNX) was synthesized via a mild, green solvent-assisted grafting strategy. The covalent incorporation of salicylaldehyde—rationally designed to leverage its aromatic and hydroxyl dual-functionality to modulate the donor–acceptor microenvironment—significantly enhanced the photocatalytic efficiency by overcoming the inherent limitations of pristine CN, such as limited visible-light utilization and rapid recombination of photogenerated charge carriers. These materials were employed for the removal of emerging organic contaminants, particularly diclofenac (DCF) and various antibiotics, from aquatic environments. The optimized catalyst, CN2.5, achieved a high 99% degradation of DCF within only 25 min under 405 nm LED irradiation, following pseudo-first-order kinetics with a reaction rate constant 1.5 times higher than that of pristine CN. Furthermore, CN2.5 demonstrated high stability and recyclability over five cycles and maintained stable performance in complex matrices, such as tap water and lake water. Mechanistic investigations revealed that the synergistic effect between salicylaldehyde and the g-C_3_N_4_ framework facilitates charge separation and promotes the generation of reactive species, primarily superoxide radicals (·O_2_^–^). This work provides valuable structural and mechanistic insights into the design of advanced g-C_3_N_4_-based materials as an effective and sustainable strategy for the efficient removal of pharmaceutical pollutants.

## Graphical abstract

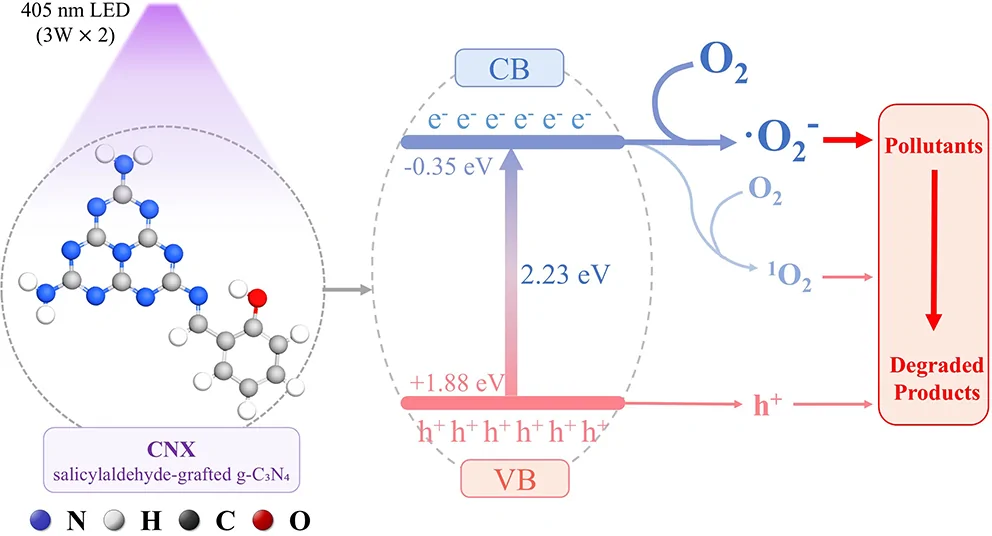

## 1 Introduction

The widespread occurrence of emerging organic contaminants (EOCs), particularly pharmaceuticals and antibiotics, in aquatic environments has become a critical global concern.^1–3^ Among these, diclofenac (DCF), a nonsteroidal anti-inflammatory drug, and various antibiotics are frequently detected in wastewater treatment plant effluents due to their inherent persistence and resistance to traditional biological treatment processes.^4–6^ These substances can accumulate in ecosystems, posing significant long-term risks to aquatic organisms and potentially facilitating the development of antibiotic-resistant bacteria. Consequently, there is an urgent need for sustainable and efficient technologies to mitigate the environmental impact of these pollutants.^7^

Harnessing solar energy for photocatalytic degradation is currently recognized as a highly sustainable approach to mitigating water pollution.^8,9^ In this context, polymeric graphitic carbon nitride (g-C_3_N_4_) stands out as an appealing metal-free candidate, benefiting from its robust physicochemical stability and an intrinsically narrow bandgap that allows for efficient visible-light harvesting.^10^ Nevertheless, the standalone performance of bulk g-C_3_N_4_ is largely constrained by intrinsic bottlenecks, notably severe charge-carrier recombination, inadequate surface area, and suboptimal solar spectrum utilization.^11,12^ To address these deficiencies, numerous tuning methodologies have been widely reported, including but not limited to morphological engineering, heteroatom incorporation, and the creation of heterostructures.^13–18^

Surface grafting represents an effective strategy to modulate the electronic structure and surface properties of g-C_3_N_4_. By covalently anchoring specific organic molecules onto the g-C_3_N_4_ framework, it is possible to enhance light-harvesting capabilities and facilitate charge carrier separation by extending the π-conjugated system through the introduction of aromatic moieties.^19^ Based on the established literature, the covalent grafting of salicylaldehyde offers a highly advantageous structural configuration compared with conventional aliphatic dopants. Its extended π-conjugated benzene ring is expected to broaden visible-light harvesting, while the electron-rich ortho-hydroxyl group can modulate local electron density, thereby constructing a localized donor–acceptor microenvironment. Furthermore, our solvent-assisted grafting provides a mild, green, and energy-efficient synthetic route. While recent advancements have successfully developed various functionalized or heterostructured carbon nitride systems for efficient pharmaceutical removal,^20,21^ the application of such specific dual-functional aromatic grafting to boost the photocatalytic degradation of recalcitrant pharmaceuticals, particularly Diclofenac (DCF), remains unexplored. To rigorously highlight this structural novelty, the performance of CN2.5 is systematically benchmarked against other highly active g-C_3_N_4_ materials (Table S2).

In this study, we report a solvent-assisted grafting strategy to synthesize salicylaldehyde-modified g-C_3_N_4_ (denoted as CNX). The incorporation of salicylaldehyde was found to induce a compact layered structure and significantly improve the optoelectronic properties of the materials. The photocatalytic activity was evaluated through the degradation of DCF and a broad spectrum of antibiotics (including Sulfamethoxazole (SMZ), Sulfamethazine (SMT), Tetracycline (TC), Chlortetracycline (CTC), and Doxycycline (DC)) under 405 nm LED irradiation. The optimized catalyst, CN2.5, demonstrated enhanced degradation efficiency and stability, even in complex water matrices like tap water and lake water. Furthermore, the underlying mechanism and the dominant role of superoxide radicals (·O_2_^–^) were systematically elucidated through scavenger experiments and photoelectrochemical characterization. This work provides new insights into the structural and mechanistic design of g-C_3_N_4_-based materials for the efficient removal of persistent organic pollutants.

## 2 Experimental Section

### 2.1 Materials and Characterization

The chemicals used and the characterization methods are detailed in the Supporting Information (Text S1, S2 and Figure S1).

### 2.2 Synthesis of g-C_3_N_4_ (CN)

Pristine graphitic carbon nitride (g-C_3_N_4_, denoted as CN) was synthesized via thermal polymerization. In a standard protocol, 0.4 g of the melamine precursor was sealed in a covered crucible and underwent thermal polycondensation inside a muffle furnace at 550 °C for 2 h. After cooling to ambient temperature naturally, the acquired pale-yellow product was thoroughly pulverized to obtain pristine CN.

### 2.3 Synthesis of Salicylaldehyde-Modified g-C_3_N_4_ (CNX)

Salicylaldehyde-modified CNX samples were prepared via a solvent-assisted grafting method. In a typical synthesis, 0.1 g of pristine CN was completely dispersed in 50 mL of anhydrous ethanol. Subsequently, different molar amounts of salicylaldehyde (*X* = 0.5, 1.5, 2.5, or 3.5 mmol) were added to the suspension. Following this, the suspension was transferred to a glass vial, sealed tightly, and magnetically stirred on a hot plate at 80 °C for 2 h to facilitate the surface reaction. After the reaction was complete and the mixture had cooled to room temperature, the resulting products were washed five times with anhydrous ethanol and distilled water, then dried at 60 °C for 12 h. The collected salicylaldehyde-modified samples were designated as CN0.5, CN1.5, CN2.5, and CN3.5. As this is a surface modification process, the solid mass recovery was nearly quantitative. The detailed schematic flowchart of this synthesis is shown in Figure S1. It should be noted that these designations reflect the nominal molar feed ratios of the salicylaldehyde precursor rather than the absolute grafted amounts. Although precise elemental quantification is hindered by adventitious surface oxygen and carbon, the progressive changes in their optical properties (such as the systematic PL quenching) effectively corroborate the increasing degree of surface functionalization.

### 2.4 Photocatalytic Activity Evaluation

Photocatalytic degradation experiments were performed in a standard 250 mL glass beaker (inner diameter about 7.0 cm). In a typical run, 20 mg of photocatalyst powder was suspended in 100 mL (0.2 g/L) of an aqueous DCF solution (20 μM, 5.92 mg/L), ensuring the methanol content in the solution remained below 1%. Prior to light exposure, the mixture underwent magnetic agitation (200 rpm) in complete darkness for 30 min to reach a stable adsorption–desorption balance. Illumination was then provided by a pair of 3 W LEDs emitting at 405 nm positioned symmetrically 7.1 cm from the breaker walls (the detailed schematic of the experimental setup is illustrated in Figure S2), providing an estimated effective illuminated area of ∼36 cm^2^. While the exact apparent quantum yield (AQY) could not be quantitatively determined due to the unavailability of an absolute optical power meter to measure the incident photon flux, the relative light-utilization efficiency of the optimized catalyst was reliably validated through rigorous parallel benchmarking against pristine CN and commercial standards under identical irradiation conditions. The reaction temperature was maintained at ambient conditions (25 ± 2 °C). At designated time points throughout the photochemical reaction, 4 mL portions of the suspension were collected. To separate the solid photocatalyst from the liquid phase, these aliquots were subjected to centrifugation (10,000 rpm for 10 min) and subsequently passed through 0.22 μm syringe filters.

The concentration of residual DCF was determined using high-performance liquid chromatography (HPLC) on an ECOM system (ECOM spol. s r.o., Prague, Czech Republic), equipped with an Agilent 1260 Infinity UV–vis detector (Agilent Technologies, Santa Clara, CA) set at 275 nm (bandwidth = 1 nm). Chromatographic separation was carried out on an Agilent Poroshell 120 SB-C_18_ column (2.1 × 100 mm, 2.7 μm) operating at a constant temperature of 30 °C. Elution was performed using an isocratic mobile phase blending acetonitrile with a 0.1% aqueous formic acid buffer (supplemented with 5.06 mM ammonium acetate) in a 30:70 volumetric ratio. The flow rate was 0.1 mL/min, with an operating pressure ranging from 56 to 60 bar. For other pollutants, including SMZ, SMT, TC, CTC, DC, and Rose Bengal (RB) (each at 20 μM), the concentrations were determined using a UV–vis spectrophotometer. The degradation percentage was calculated using eq 1

1 $$\mathrm{Degradation efficiency}\left(\%\right)=\eta =\frac{C_{0}-C}{C_{0}}\times 100\%$$

Where η represents the degradation efficiency (%), and *C*_0_ and *C* represent the initial molar concentration (strictly defined as the concentration after the 30 min dark adsorption equilibrium) and the final molar concentration, respectively.

Degradation intermediates were analyzed using electrospray ionization-mass spectrometry (ESI-MS; Shimadzu, MS-8060).

### 2.5 Scavenger Experiments and Real Water Sample Pretreatment

To elucidate the primary reactive species involved in the degradation process, scavenger experiments were conducted under conditions identical to those of the photocatalytic test, with the addition of specific scavengers prior to illumination. The stock solutions of scavengers, including isopropanol (IPA), sodium nitrate, ammonium oxalate (AO), l-histidine (l-His), and *p*-benzoquinone (p-BQ) were preprepared at a concentration of 0.1 M. Before turning on the light source, predefined volumes of the respective stock solutions were introduced into the reactant mixtures. This established a uniform working concentration of 5 mM, strategically designed to quench specific reactive intermediates, namely hydroxyl radicals (·OH), electrons (e^–^), holes (h^+^), singlet oxygen (^1^O_2_), and superoxide radicals (·O_2_^–^). Furthermore, to evaluate the degradation efficiency in practical environments, real water samples including tap water and lake water were collected and pretreated. Tap water was obtained from the analytical laboratory at National Taitung University (25.6 °C, pH 8.18), while lake water was collected from Jingxin Lake on the university campus (25.1 °C, pH 9.23). All environmental water matrices were initially pretreated by centrifugation (10,000 rpm, 10 min) and subsequent filtration via a 0.22 μm membrane. This crucial step eliminated particulates and large organic impurities that could otherwise disrupt the subsequent photocatalytic evaluations.

### 2.6 Photoelectrochemical Measurements

All photoelectrochemical tests were carried out in a conventional three-electrode configuration. This setup utilized an Ag/AgCl reference electrode, a Pt wire auxiliary electrode, and the sample-deposited FTO glass as the working electrode, which were immersed in a neutral 0.5 M Na_2_SO_4_ electrolyte (pH = 6.87). To fabricate the working electrode, an ethanolic dispersion containing 20 mg of the catalyst and 10 μL of a Nafion binder (5 wt %) was ultrasonicated for 30 min to yield a homogeneous slurry. This mixture was subsequently drop-cast onto a precleaned FTO substrate (2 × 2 cm^2^) and left to dry overnight at 60 °C.

## 3 Results and Discussion

### 3.1 Structural and Chemical Characterization

The morphological characteristics of the CN and CNX samples were investigated by scanning electron microscopy (SEM). As illustrated in Figure 1a, the pristine CN sample exhibited a typical layered structure with relatively loose sheets. In comparison, the grafting of salicylaldehyde triggered significant morphological alterations in the CNX samples (Figure 1b–e). The modified CNX exhibited a fragmented morphology, characterized by increased surface roughness and structural defects. This structural evolution suggests that the salicylaldehyde modification effectively alters the layered stacking structure.

**Figure 1:**
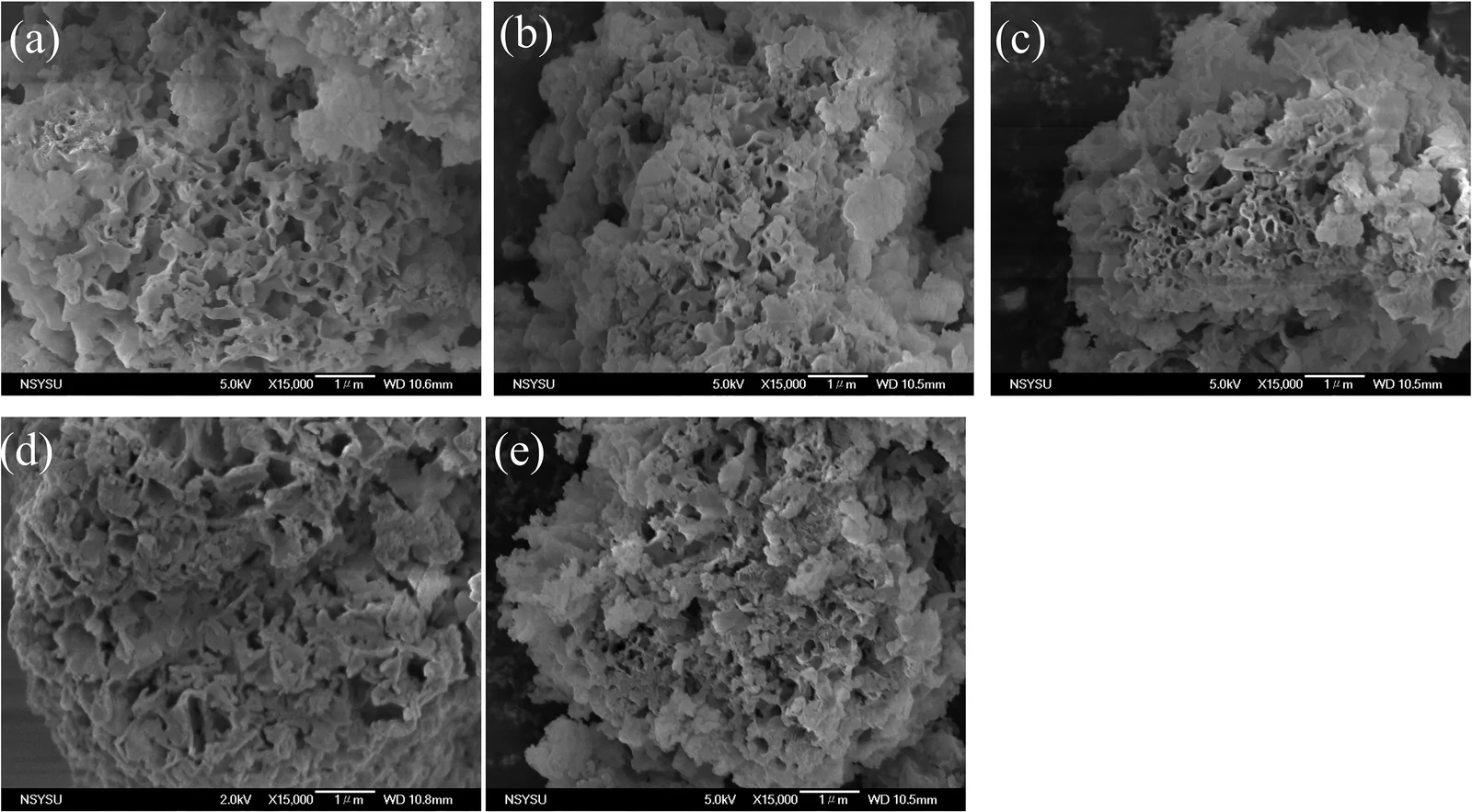
SEM images of (a) CN, (b) CN0.5, (c) CN1.5, (d) CN2.5, and (e) CN3.5 samples at a magnification of ×15k. The scale bar in all images represents 1 μm.

According to the XRD profiles (Figure 2a), all synthesized materials display two distinct reflection signals near 13.0 and 27.1°. These correspond to the (100) and (002) crystallographic planes, reflecting the in-plane packing of the tri-s-triazine motifs and the periodic interlayer stacking of the conjugated sheets, respectively.^22–24^ These peaks are characteristic of the typical in-plane structural ordering and interlayer stacking of the g-C_3_N_4_ framework. Notably, the (002) peak of the CNX samples exhibited a significant shift toward higher angles (from 27.1 to 27.5°). This contraction of the interlayer spacing, coupled with the fragmented morphology observed in SEM, indicates that the modification effectively modulates the layered stacking, leading to a structurally modified framework.

**Figure 2:**
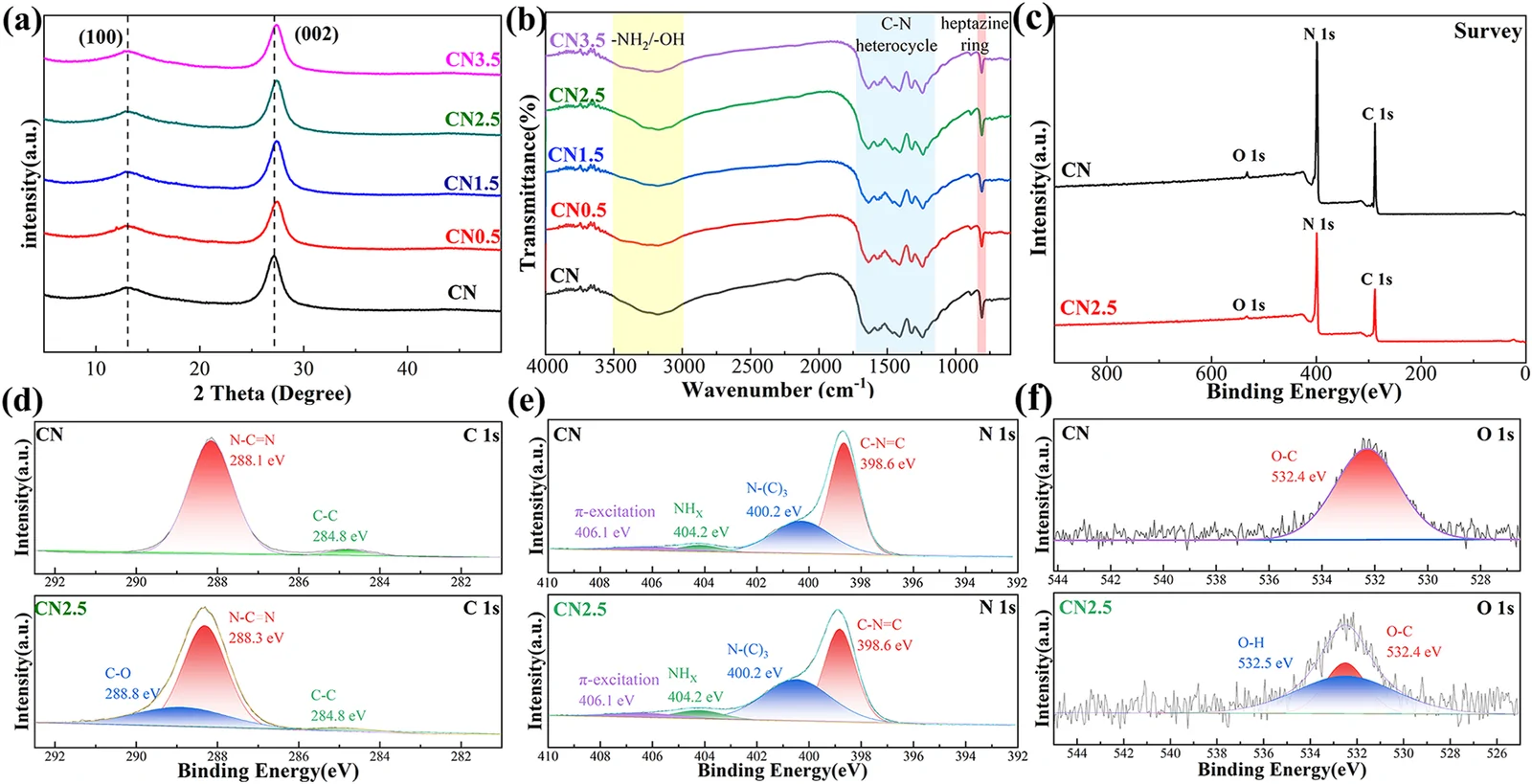
(a) XRD and (b) FTIR for CN, CN0.5, CN1.5, CN2.5, and CN3.5. High-resolution XPS spectra of (c) survey spectra, (d) C 1s, (e) N 1s, and (f) O 1s.

Fourier transform infrared (FTIR) spectra revealed a broad absorption region between 3000 and 3500 cm^–1^, originating from the stretching modes of terminal −NH_2_/–OH functionalities.^25,26^ The fingerprint region from 1200 to 1650 cm^–1^ is populated by the skeletal stretching vibrations of the C–N and C=N heterocycles,^27^ while the distinct resonance at 810 cm^–1^ is attributed to the breathing mode of the triazine rings.^28^ The characteristic peaks of CN and CNX remained largely consistent, indicating that the framework structure was preserved and the overall composition remained stable after modification.

X-ray photoelectron spectroscopy (XPS) was performed to confirm the successful grafting of salicylaldehyde onto the CN2.5 catalyst. The elemental composition was initially surveyed via XPS, confirming that the materials dominantly consist of carbon, nitrogen, and oxygen. As shown in Figure 2d, the high-resolution C 1s spectra exhibited peaks at 284.8 and 288.1 eV, assigned to C–C and N–C=N bonds,^27,29,30^ respectively. Additionally, a new peak appeared in the CN2.5 sample at 288.8 eV, which is attributed to the C–O bond.^31^ This aligns with the presence of the ortho-hydroxyl group (−OH) in the salicylaldehyde molecule, confirming that the salicylaldehyde was successfully bonded to the carbon atoms. Figure 2e presents the high-resolution N 1s spectra, which exhibit four distinct peaks at 398.6, 400.2, 404.2, and 406.1 eV, assigned to N=C, N-(C)_3_, C-NH_*x*_, and π-excitation bonds,^28,32^ respectively. In the O 1s spectra (Figure 2f), the pristine CN showed a single peak at 532.4 eV corresponding to O–C.^28^ For the modified CN2.5 sample, the O 1s peak envelope encompasses overlapping contributions from both the pristine matrix and the newly introduced O–C and O–H groups from the salicylaldehyde moiety. Although the binding energies of these oxygen environments are closely spaced (∼532.4–532.5 eV) near the instrumental resolution limit, the noticeable change in the O 1s region—complemented by the clear appearance of −OH stretching vibrations in the FTIR spectra (Figure 2b)—qualitatively substantiates the successful chemical anchoring of salicylaldehyde. The plausible chemical pathway for this anchoring process via Schiff-base condensation is schematically proposed in Figure S3. Importantly, this green, catalyst-free synthetic route relies on the intrinsic solid-base properties of the g-C_3_N_4_ framework and the high reactivity of salicylaldehyde, which is highly feasible under mild ethanol reflux and has been widely validated by recent literature.^19,33,34^

### 3.2 Optoelectronic Property

The optical properties and electronic band structures were systematically investigated using UV–vis diffuse reflectance spectroscopy (UV–vis DRS). As shown in Figure 3a, the DRS results demonstrate that all materials display strong intrinsic absorption between 200 and 400 nm, driven by the characteristic π–π* transitions of the conjugated carbon nitride network. Compared to the pristine CN, the salicylaldehyde-modified samples exhibited a red shift in their absorption edges. Notably, the CN2.5 sample exhibited the strongest visible-light absorption, indicating the most efficient utilization of photon energy. The bandgap energies (*E*_g_) were determined using Tauc plots (Figure 3b), yielding a value of 2.26 and 2.23 eV for CN and CN2.5, respectively. This decrease indicates that the grafting of functional groups modulates the electronic structure and facilitates the excitation of photogenerated charge carriers under visible light.^35–39^

**Figure 3:**
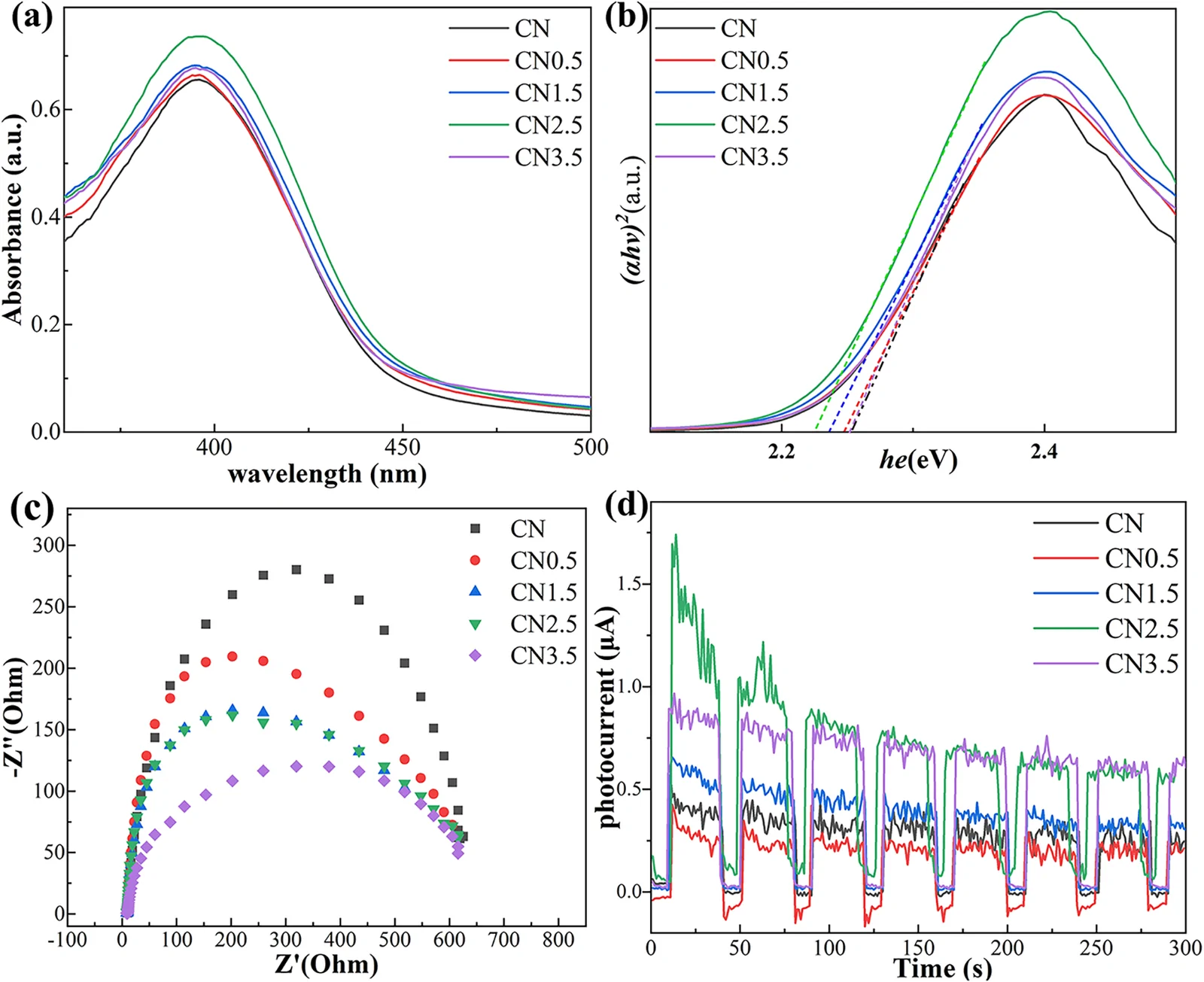
(a) UV–vis DRS spectra, (b) Tauc plot, (c) EIS plots of CN and CNX samples, and (d) transient photocurrent response curves.

To investigate the separation and transport of photogenerated charges, electrochemical impedance spectroscopy (EIS) and transient photocurrent response measurements were conducted. In EIS Nyquist plots, a contracted arc radius intuitively signifies a lower interfacial charge-transfer resistance between the electrode surface and the electrolyte. As shown in Figure 3c, CN3.5 and CN2.5 exhibited smaller interfacial resistance values compared to pristine CN. Furthermore, CN2.5 exhibited the highest photocurrent density (Figure 3d), indicating the highest population of photogenerated electrons and optimal carrier migration efficiency.

To systematically investigate the separation and recombination behaviors of photogenerated charge carriers, steady-state photoluminescence (PL) measurements were performed. As displayed in Figure 4a, both pristine CN and the modified materials exhibit a distinct emission peak at 405 nm (excitation wavelength of 390 nm). Notably, the modified samples (particularly CN2.5) show a significantly quenched PL intensity compared to pristine CN. This severe PL quenching strongly indicates that the chemical anchoring of salicylaldehyde effectively suppresses radiative recombination of electron–hole pairs, thereby establishing an efficient intramolecular electron-transfer pathway within the donor–acceptor-engineered framework.

**Figure 4:**
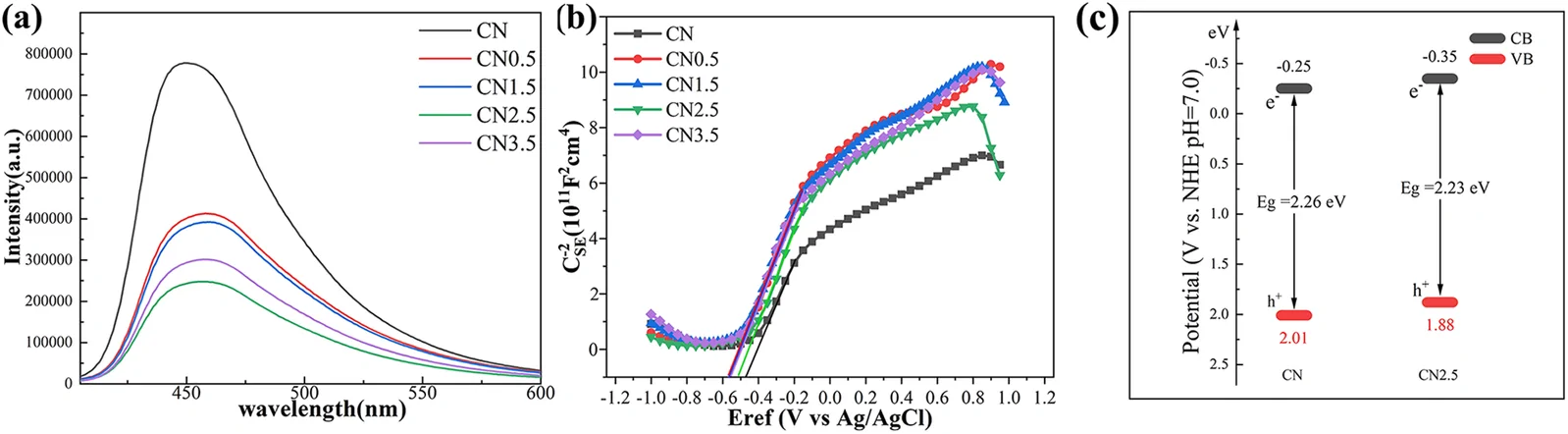
(a) PL of CN, CN0.5, CN1.5, CN2.5, and CN3.5 samples, (b) electronic band structures of CN and CN2.5, and (c) Mott–Schottky plots.

Subsequently, to further clarify the electronic band alignments and thermodynamic feasibility of the reactive species, Mott–Schottky measurements were conducted; flat-band potentials *E*_fb_ were derived via Mott–Schottky analysis (Figure 4b). For standard n-type semiconductor materials, these measured *E*_fb_ values provide a reliable estimation of the conduction band minimum. The measured *E*_fb_ values (vs Ag/AgCl) for CN, CN0.5, CN1.5, CN2.5, and CN3.5 were −0.45, −0.55, −0.55, −0.55, and −0.50 eV, respectively. Importantly, as specified in Section 2.6, these electrochemical measurements were conducted in the electrolyte at a precise pH of 6.82. These values were converted to the normal hydrogen electrode (NHE) scale using eq 2

2 E(NHE)=E(Ag/AgCl)+0.197V

Consequently, the CB edge potential of the optimized CN2.5 sample at pH 6.82 was determined to be −0.35 V vs NHE. From a thermodynamic perspective, this CB position is substantially more negative than the standard redox potential required for the single-electron reduction of oxygen (O_2_/·O_2_^–^ = −0.18 V vs NHE).^40^ This energy alignment ensures a strong thermodynamic driving force for the efficient and spontaneous generation of superoxide radicals (·O_2_^–^), which perfectly corroborates the dominant role of ·O_2_^–^ verified in our scavenger experiments. By combining these electrochemical results with the bandgap energies (*E*_g_) obtained from UV–vis DRS, the electronic band structure was constructed, as illustrated in Figure 4c.

### 3.3 Photocatalytic Degradation

The photocatalytic performance of the synthesized materials was evaluated by monitoring the degradation of DCF under 405 nm LED irradiation (Figure 5a), with residual concentrations precisely quantified by HPLC using the established calibration curve (Figure S4). Control experiments confirmed that no significant degradation of DCF occurred via direct photolysis without a catalyst (Blank), and no further degradation was observed in the presence of the catalyst without light irradiation. The optimized catalyst, CN2.5, demonstrated high catalytic activity, achieving over 99% degradation of DCF within 25 min. Intriguingly, despite the morphological fragmentation observed in SEM, dark adsorption tests (Figure S5) revealed that CN2.5 exhibited a lower adsorption capacity (7%) compared to pristine CN (18%). This phenomenon is likely attributed to the grafting of salicylaldehyde, which may occupy specific surface active sites or alter the surface charge density, thereby reducing physical adsorption. Nevertheless, this emphasizes that the improved performance of CN2.5 is predominantly driven by its enhanced photocatalytic activity rather than simple adsorption processes.

**Figure 5:**
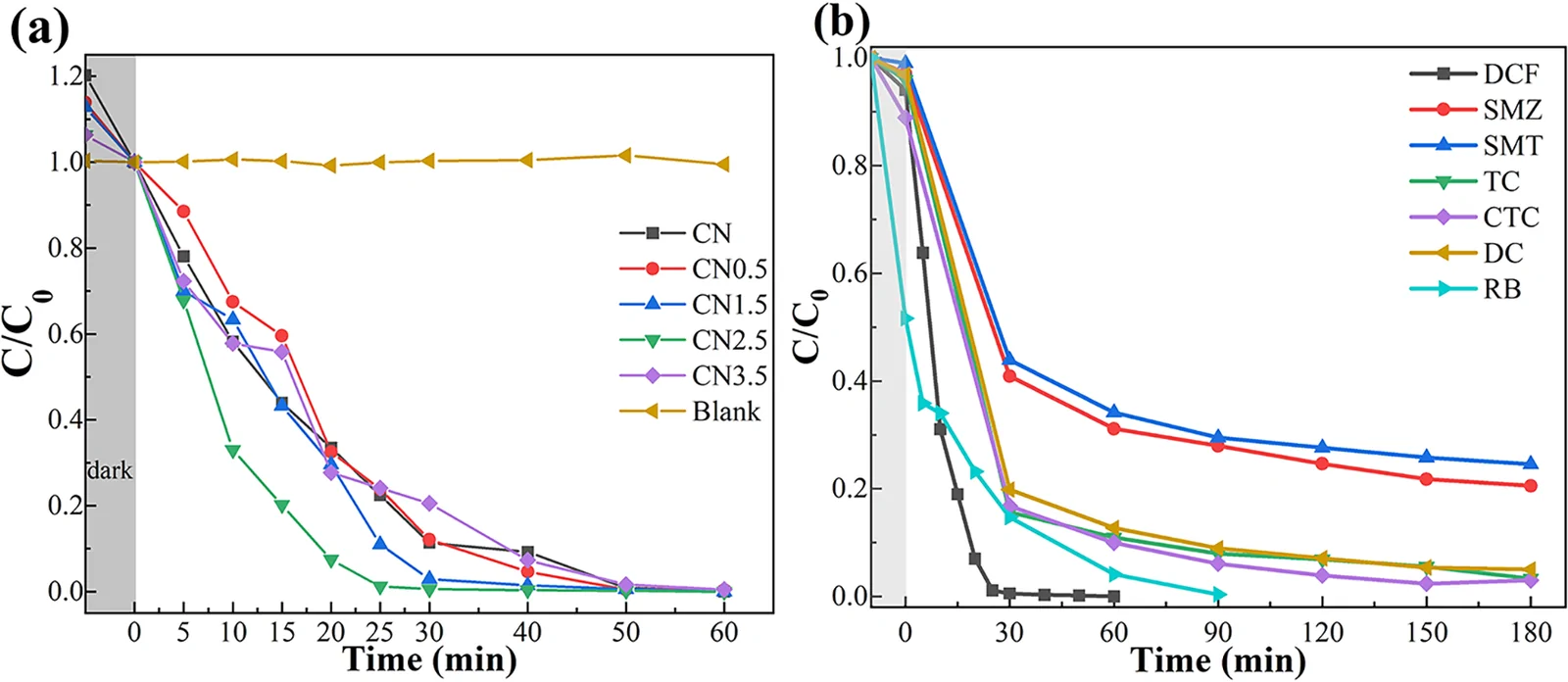
(a) Time-dependent DCF removal profiles for pristine CN and modified CNX catalysts during 405 nm LED exposure. (b) Broad-spectrum photocatalytic elimination performance of CN2.5 against various antibiotics and the RB dye.

The degradation kinetics followed a pseudo-first-order rate law (*−*ln­(*C*/*C*_0_) = *kt*, Figure S6). The kinetic fitting exhibited strong linear correlation (*R* ^2^ = 0.9769 for the optimized CN2.5), demonstrating high analytical reliability. Specifically, the apparent reaction rate constant *k* for CN2.5 was determined to be 0.1282 min^–1^, which is 1.5 times higher than that of pristine CN (*k* = 0.0858 min^–1^), further confirming its enhanced efficiency. Furthermore, the versatility of the catalyst was evaluated against various pollutants, including antibiotics (SMZ, SMT, TC, CTC, DC) and organic dyes (RB) (Figure 5b). Within 180 min, high degradation rates (94.99–96.98%) were achieved for all tested antibiotics, while RB was completely removed after 90 min. These results highlight the broad potential of CN2.5 for broad-spectrum environmental remediation

### 3.4 Photodegradation Mechanism and Scavenger Studies

To identify the primary reactive oxygen species (ROS) responsible for DCF degradation, scavenger experiments were performed (Figure 6). The addition of scavengers for hydroxyl radicals (·OH), electrons (e^–^), holes (h^+^), singlet oxygen (^1^O_2_), and superoxide radicals (·O_2_^–^) significantly impacted the degradation efficiency. The substantial inhibition observed upon adding p-BQ indicates that ·O_2_^–^ plays a dominant role in the overall reaction. Furthermore, h^+^ and ^1^O_2_ were identified as key active species. We contend that the synergistic degradation mechanism is fundamentally governed by the improved optoelectronic properties. Specifically, the extended π–conjugated system from covalent salicylaldehyde grafting ensures efficient charge separation (as previously evidenced by PL and photocurrent analyses), while the highly negative CB edge (−0.35 V vs NHE) provides ample thermodynamic driving force for the single-electron reduction of O_2_ (−0.18 V vs NHE).^40^ This energetic alignment directly accounts for the dominant role of ·O_2_^–^ verified in the scavenger experiments. In summary, the degradation of DCF by CN2.5 relies on the synergistic action of ·O_2_^–^, h^+^, and ^1^O_2_.

**Figure 6:**
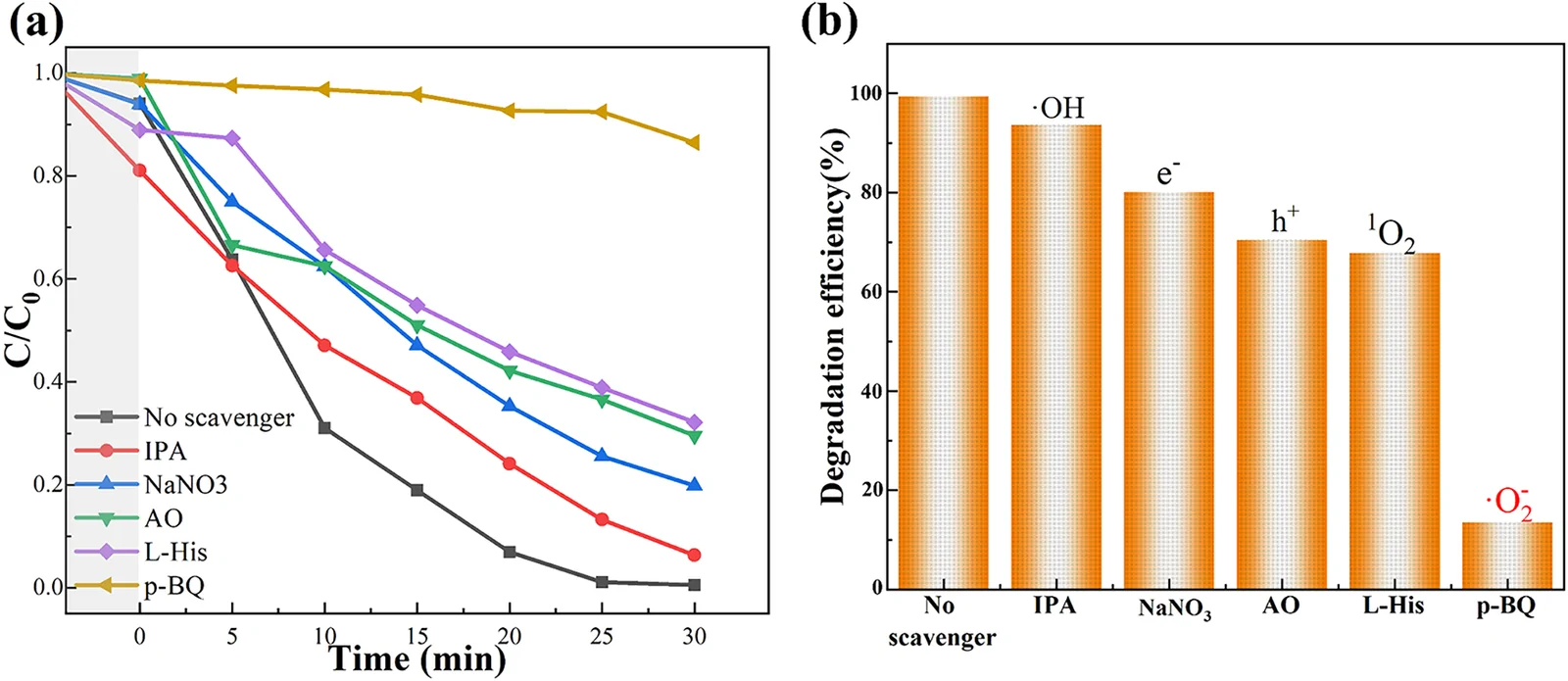
(a) Kinetic curves and (b) corresponding removal efficiencies for DCF degradation using CN2.5 in the presence of distinct radical scavengers (each introduced at a 5 mM concentration) to identify the dominant active species.

### 3.5 Reaction Intermediates and Degradation Pathway

Based on MS analyses (Figure S7), potential degradation intermediates of DCF were identified, and a plausible degradation pathway is proposed (Figure S8). The main degradation steps appear to involve dechlorination, dehydration, and hydroxylation reactions. The initial steps lead to three primary intermediates: P1 (via combined dechlorination/hydroxylation), P2 (via full dechlorination), and P3 (via dehydration and cyclization).^40,41^ Following these routes, the cleavage of the C–N bond in P1 yields the single-ring fragment P5, while the direct decarboxylation of P2 forms P6.^41,42^ Concurrently, the lactam P3 undergoes sequential oxidation to form P4, which further fragments into smaller single-ring derivatives such as P9 and P10.^40,42^ Furthermore, the deep oxidation of P2 and P4 leads to the highly conjugated isobaric intermediates P7 and P8 (Mw 223 isomers), respectively.^40,41^ Alternatively, nondechlorinated oxidation pathways generate quinone-like products P11 (Mw 325), which ultimately undergo direct ring-opening to form small molecular compounds.^40–42^

T.E.S.T. software predictions (summarized in Table S1) reveal that while the parent DCF and several early stage intermediates retain high acute toxicity (LC50 < 10 mg/L), the deep oxidation process successfully transforms them into less toxic ’harmful’ category compounds (LC50 10–100 mg/L), such as P3, P5, P9, and P10. This confirms that the proposed photocatalytic pathway effectively mitigates the overall environmental toxicity of the pharmaceutical pollutants.

### 3.6 Stability and Practical Application Evaluation

The stability and reusability of a photocatalyst are critical parameters for its potential industrial implementation. In this study, the operational stability of the optimized CN2.5 was assessed through five consecutive photocatalytic cycles for the degradation of DCF. As illustrated in Figure 7, the photocatalytic efficiency remained remarkably high, with only a marginal decrease of 6.8% observed from the first to the fifth cycle. This minimal loss in activity is likely attributed to the slight mass loss of the catalyst during the recovery and washing steps rather than structural degradation. Although direct postreaction morphological and structural characterizations were not performed, the remarkably consistent degradation efficiency across consecutive cycles strongly implies that the photocatalyst’s structural and morphological integrity is well preserved. Any significant framework collapse, structural degradation, or leaching of active sites would have inevitably resulted in a severe decline in photocatalytic performance during the recycling tests. Furthermore, this highly consistent performance across independent runs inherently validates the high reproducibility and analytical reliability of our experimental setup. These results demonstrate that CN2.5 possesses high structural stability and recyclability, meeting the requirements for sustainable environmental remediation.

**Figure 7:**
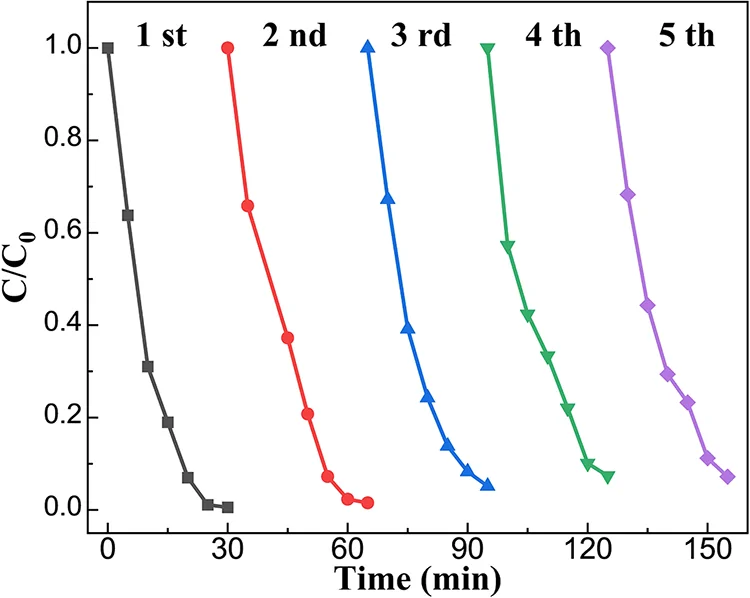
Cycling test of CN2.5 for photocatalytic degradation of DCF under visible-light irradiation.

Furthermore, the performance of photocatalysts in real-world scenarios is often hindered by complex water matrices containing various inorganic ions and organic matter. To evaluate the practical applicability of CN2.5, experiments were conducted using municipal tap water and natural lake water as reaction media (Figure 8a). Notably, the CN2.5 catalyst maintained stable photocatalytic activity across these different water samples. In the lake water matrix, a degradation efficiency of 97.8% was achieved within only 15 min, corresponding to a high reaction rate constant of *k* = 0.2395 min^–1^. Elevated removal rates in natural water matrices, compared to ultrapure water, have been similarly observed in recent studies.^40^ Although rigorous water composition analysis is needed to pinpoint the exact mechanism, this catalytic boost is theoretically attributed to indigenous dissolved organic compounds or specific trace ions. These constituents potentially function as naturally occurring photosensitizers or trigger the generation of supplementary radical species, thereby accelerating the overall decomposition kinetics. In the tap water matrix, although the reaction rate was slightly lower due to potential interference, the degradation still reached 96.6% within 70 min (*k* = 0.052 min^–1^). These findings underscore CN2.5′s high resistance to environmental interference and its high potential for efficient treatment in complex aquatic environments. While this proof-of-concept study primarily establishes the structural advantages and fundamental mechanisms of the catalyst, systematic investigations into the effects of pH and ionic strength will be a key focus of our future studies to further optimize its real-world implementation.

**Figure 8:**
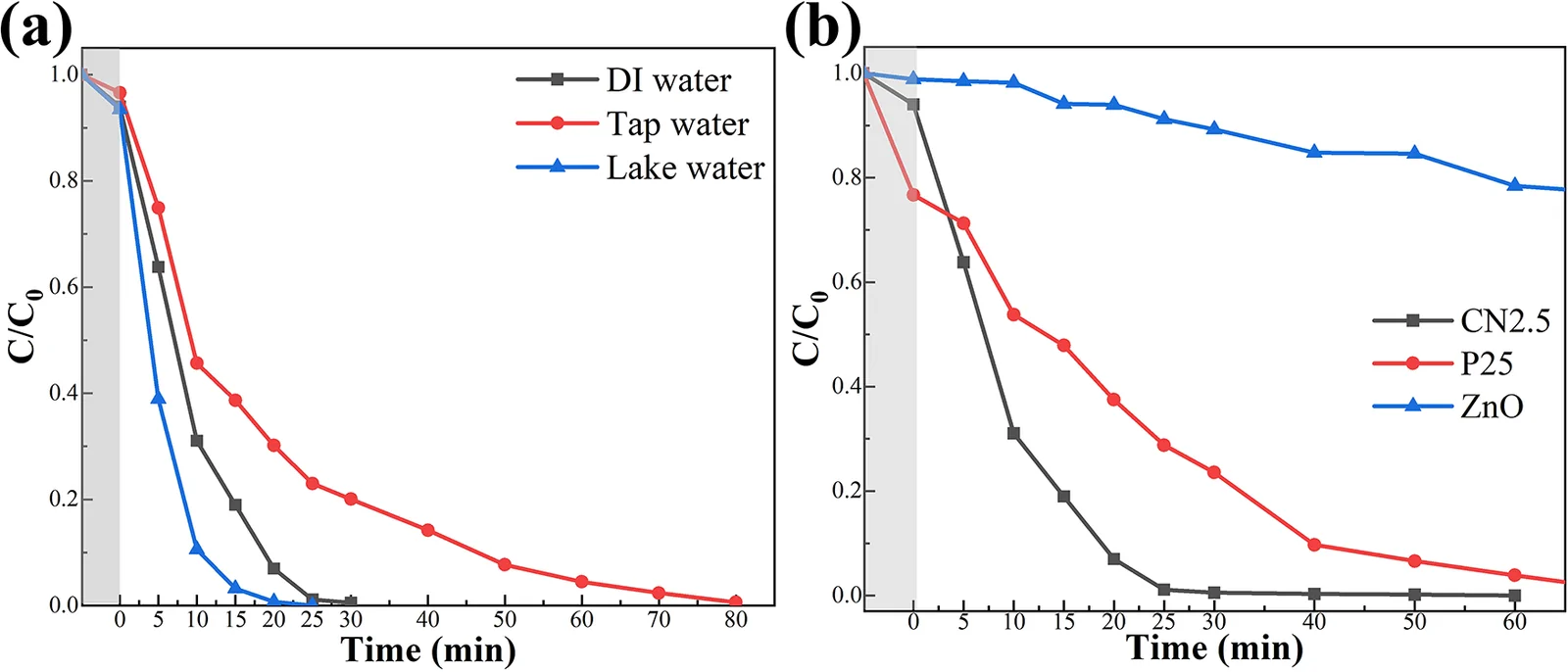
(a) Photocatalytic degradation of DCF in DI water, tap water, and lake water by CN2.5, and (b) comparison of DCF degradation using commercial CN2.5, P25, and ZnO under two 3 W 405 nm LEDs.

To further benchmark their performance, commercial P25 and ZnO were also evaluated under identical conditions of 405 nm LED irradiation (Figure 8b). As expected from their wide bandgaps (∼3.2 eV) and strict dependence on UV light, both traditional benchmarks exhibited inferior photocatalytic activity toward DCF degradation under this visible-light excitation. This stark contrast highlights the critical necessity of developing visible-light-responsive materials like CNX. Furthermore, to provide a fair and highly rigorous evaluation of our optimized catalyst, the performance of CN2.5 was benchmarked against other recently reported visible-light-driven g-C_3_N_4_-based photocatalysts. As systematically summarized in Table S2, CN2.5 exhibits highly competitive degradation efficiency and kinetics even under mild conditions (accounting for catalyst dosage and initial pollutant concentration), underscoring the remarkable advantage of the salicylaldehyde grafting strategy.

## 4 Conclusions

In this study, a salicylaldehyde-grafting strategy was successfully implemented to significantly enhance the structural and optoelectronic properties of graphitic carbon nitride (g-C_3_N_4_). Characterization results revealed that the modified CNX materials exhibited a highly fragmented layered structure with increased surface defects and enhanced visible-light-harvesting capabilities, effectively promoting the separation and migration of photogenerated charge carriers. Photoelectrochemical tests confirmed that the optimized CN2.5 exhibited the highest photocurrent density and the lowest interfacial charge-transfer resistance among all samples.

Under 405 nm LED irradiation, CN2.5 demonstrated high degradation activity toward DCF, achieving a 99% removal rate within 25 min. Its pseudo-first-order rate constant (*k* = 0.1282 min^–1^) was 1.5 times higher than that of pristine CN and significantly outperformed the commercial P25 and ZnO benchmarks. Mechanistic investigations identified superoxide radicals (·O_2_^–^) as the primary reactive species driving the degradation process. Furthermore, CN2.5 exhibited high cycling stability (only a 6.8% decrease after five cycles) and strong potential for practical application, achieving a 97.8% removal rate in lake water within 15 min. This research provides structural and mechanistic insights into the design of advanced g-C_3_N_4_-based materials, demonstrating that salicylaldehyde modification is an effective and sustainable strategy for the photocatalytic elimination of persistent pharmaceutical contaminants.

## Supplementary Material



## Notes

Detailed descriptions of materials and characterization methods; schematic illustrations of the synthesis and photocatalytic setup; proposed Schiff-base condensation mechanism; HPLC chromatograms and standard calibration curves; dark adsorption evaluation; kinetic modeling plots; MS spectra with the proposed degradation pathway; toxicity assessments of the parent compound and intermediates; and a systematic literature comparison of photocatalytic performance (PDF)
