# Supplementary material for: Salicylaldehyde-Grafted g‑C3N4 for Enhanced Photocatalytic Degradation of Diclofenac and Antibiotics
Source: ACS Omega. 2026 Sep 25;11(39):59583–92. doi: 10.1021/acsomega.6c06050 (PMC13647902; doi:10.1021/acsomega.6c06050)
Supplement: Supplementary file 1 [file ao6c06050_si_001.pdf]

Supporting Information

Salicylaldehyde-Grafted g-C_3N_4 for Enhanced Photocatalytic Degradation of Diclofenac and Antibiotics

Yu-Shan Lin, Yu-Shen Lin, I-Cheng Liu, Tai-Chia Chiu, Cho-Chun Hu**

Department of Applied Science, National Taitung University, No. 369, Sec. 2, University Road, Taitung City, Taitung County 95092, Taiwan (R.O.C.)

Corresponding Author

*Department of Applied Science, National Taitung University, No. 369, Sec. 2, University Road, Taitung City, Taitung County 95092, Taiwan (R.O.C.)

Tel.: +886 (089)517991#6434.

E-mail address: simon@kaishinedu.com (Y.S. Lin), cchu@nttu.edu.tw (C.C. Hu)

Text S1. **Materials.** Melamine, urea, salicylaldehyde, diclofenac sodium salt (DCF), sulfamethazine (SMZ), sulfamethoxazole (SMT), tetracycline (TC), chlortetracycline (CTC), doxycycline (DC), rhodamine B (RB), zinc oxide, titanium dioxide (TiO_2) P25 nanoparticles, and ammonium acetate were purchased from Sigma-Aldrich (St. Louis, MO, USA), sodium nitrate, ammonium oxalate monohydrate (AO), L-histidine (L-His), 1,4-benzoquinone (p-BQ), ammonium ethanoate and sodium sulfate were purchased from Sigma-Aldrich (St. Louis, United States). Formic acid was purchased from Honeywell (Charlotte, NC, USA). Methanol was purchased from Spectrum Chemical (New Brunswick, NJ, USA). Acetonitrile (ACN) was purchased from J. T. Baker (Phillipsburg, NJ, USA). Nafion perfluorinated resin was purchased from TRC (Toronto, ON, Canada). Deionized (DI) water was used throughout the experiments. All chemicals were used as received without further purification.

Text S2. Characterization. Sample morphologies were examined using a field emission scanning electron microscope (FESEM; JEOL JSM-7800F). Powder X-ray diffraction (XRD) patterns were recorded using a Low-Temperature XRD (Bruker, D8 Discover X-ray Diffraction System). X-ray Photoelectron Spectrometer (XPS) was performed on Thermo Scientific K-Alpha. Binding energies were calibrated using the adventitious C 1s peak at 284.8 eV. UV-vis diffuse reflectance spectra (DRS) were obtained using a Specord 210 plus UV-vis spectrophotometer (Analytik Jena, Germany). Photoluminescence (PL) spectra were recorded on an RF-6000 fluorescence spectrometer (Shimadzu, Japan) using an excitation wavelength of 380 nm.

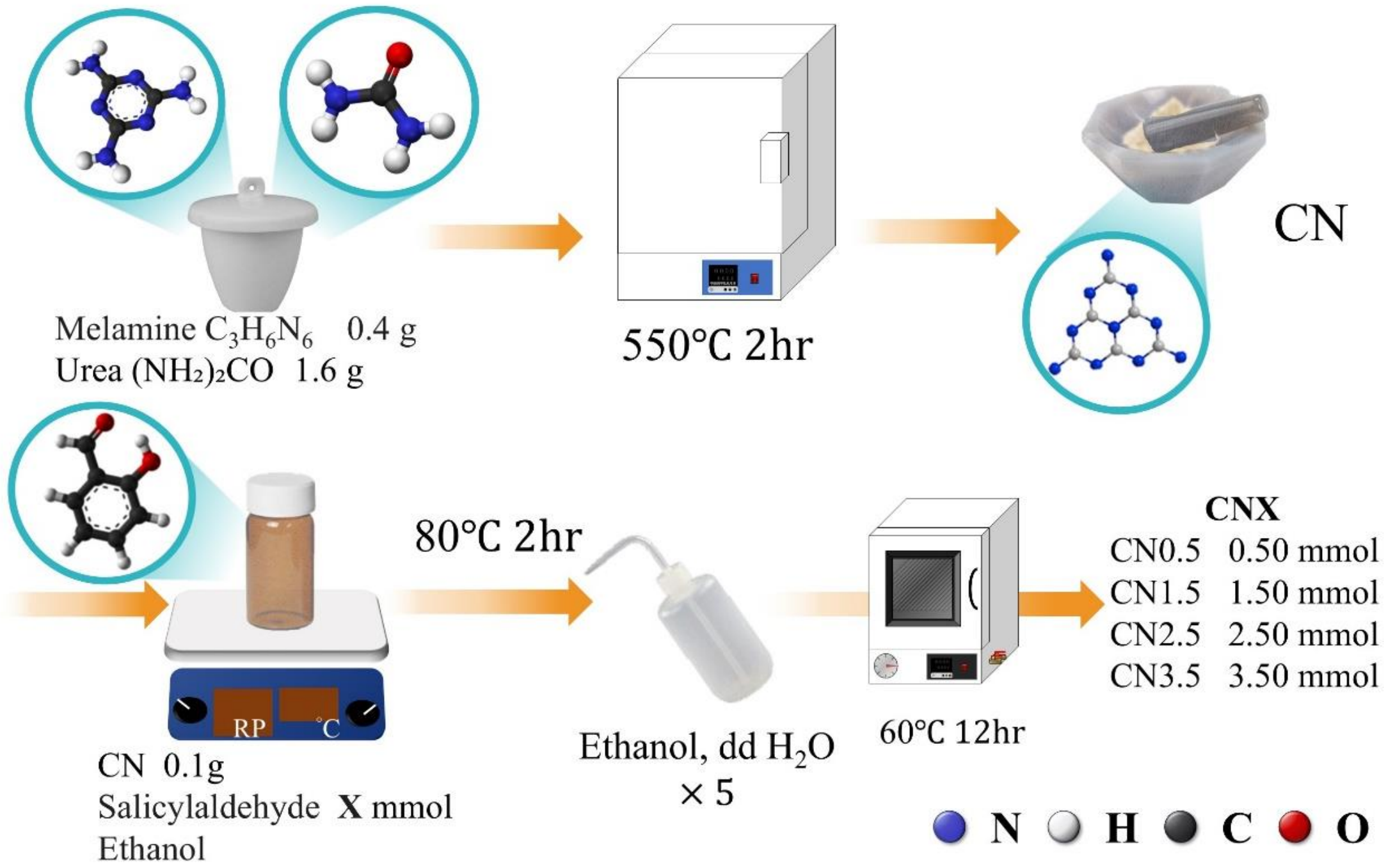

Figure S1. Schematic illustration of g-C_3N_4 synthesis.

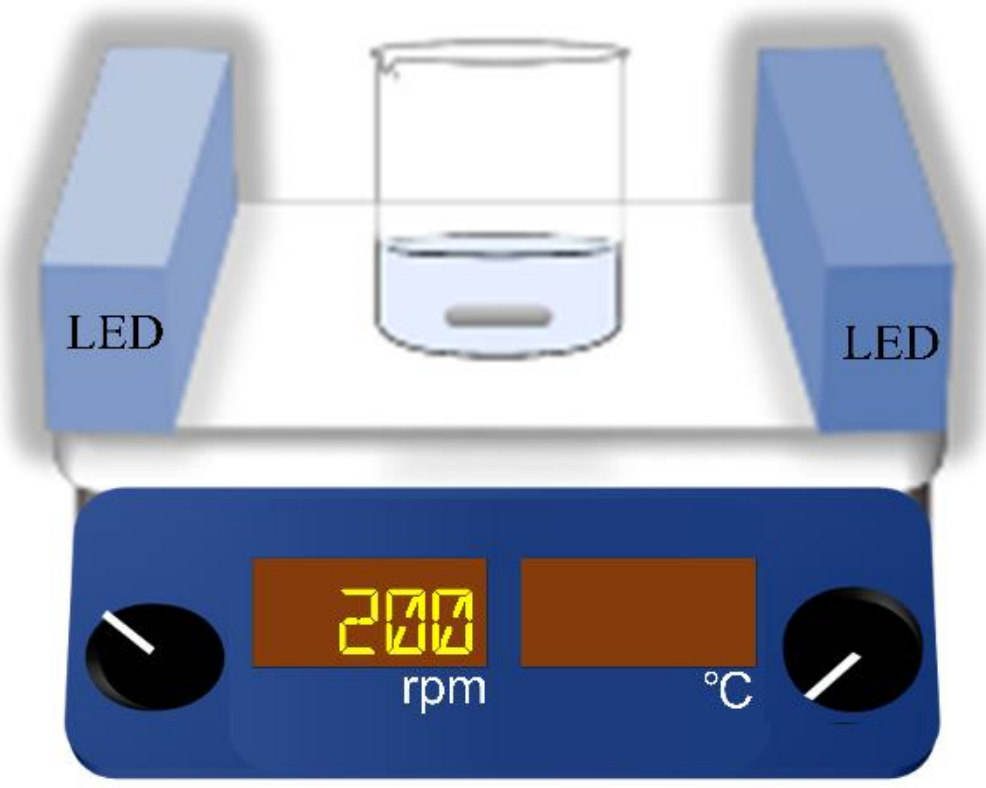

Figure S2. Schematic illustration of the photocatalytic experimental setup. Two 3 W 405 nm LED light sources were positioned symmetrically at a distance of 7.1 cm from the walls of a 250 mL glass beaker containing 100 mL of the reaction suspension. The effective illuminated area is estimated to be ~36 cm². The reaction temperature was maintained at ambient conditions (25 ± 2 °C)

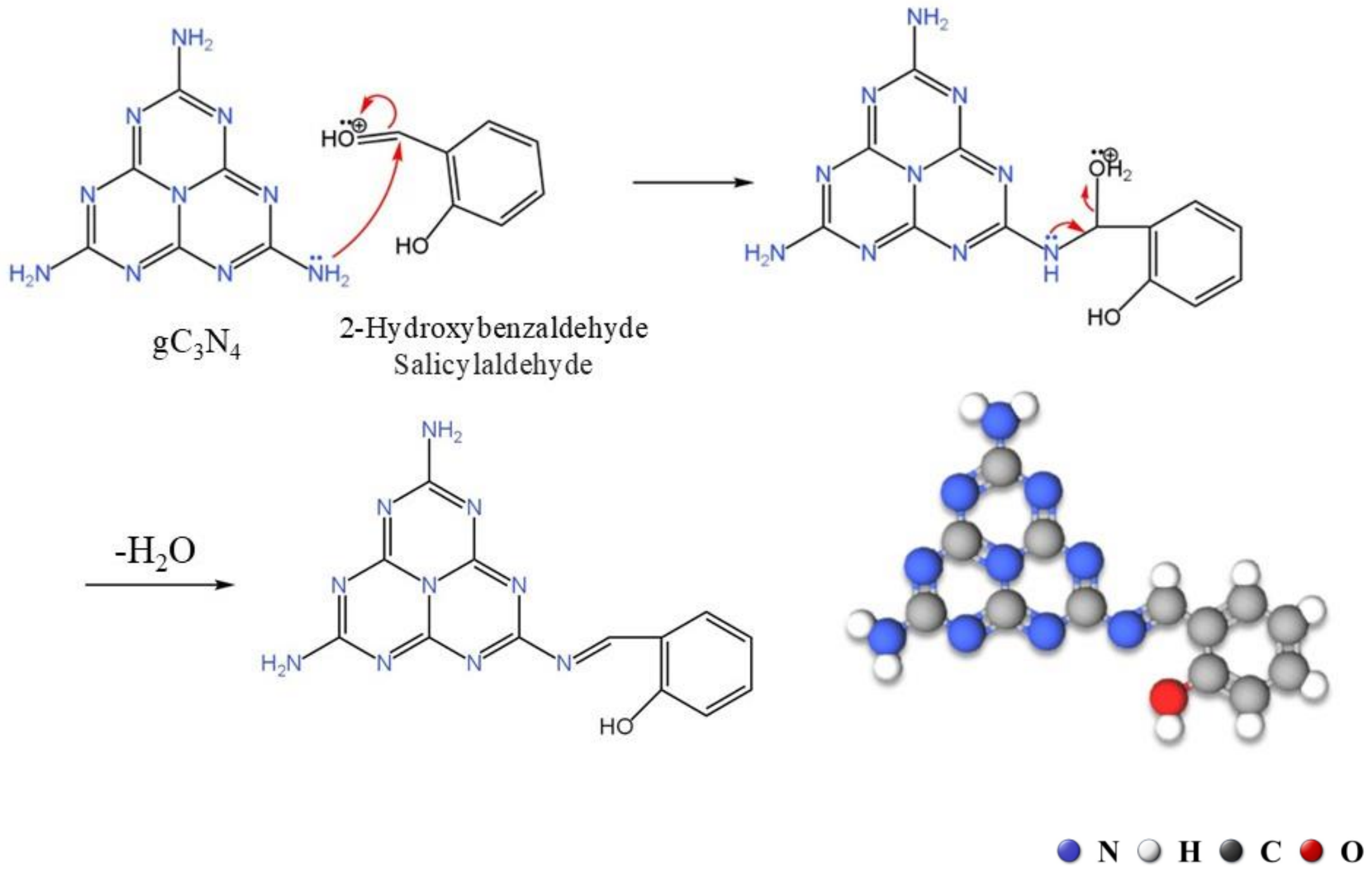

Figure S3. Proposed mechanism for the chemical anchoring of salicylaldehyde onto the g-C_3N_4 framework via Schiff-base condensation.

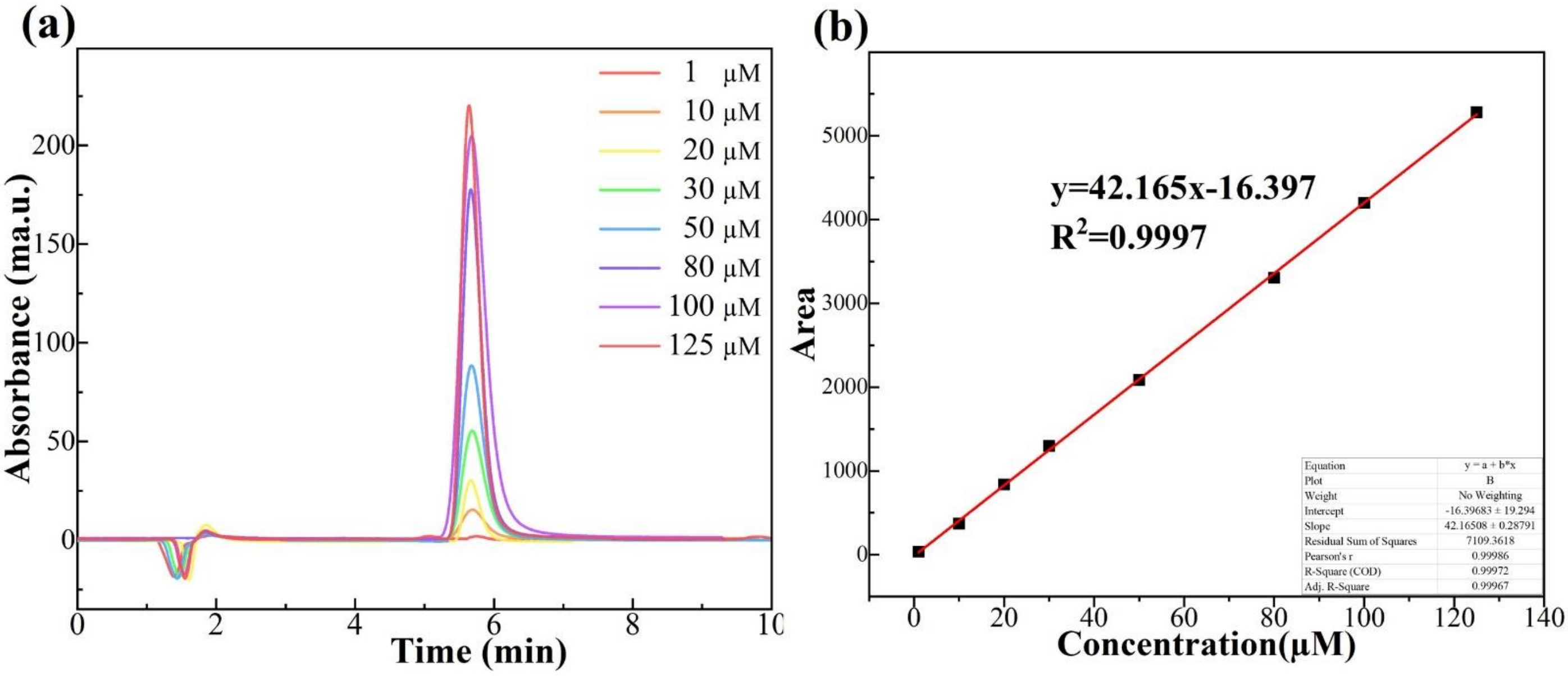

Figure S4. The (a) HPLC chromatogram and (b) standard curve of DCF concentration.

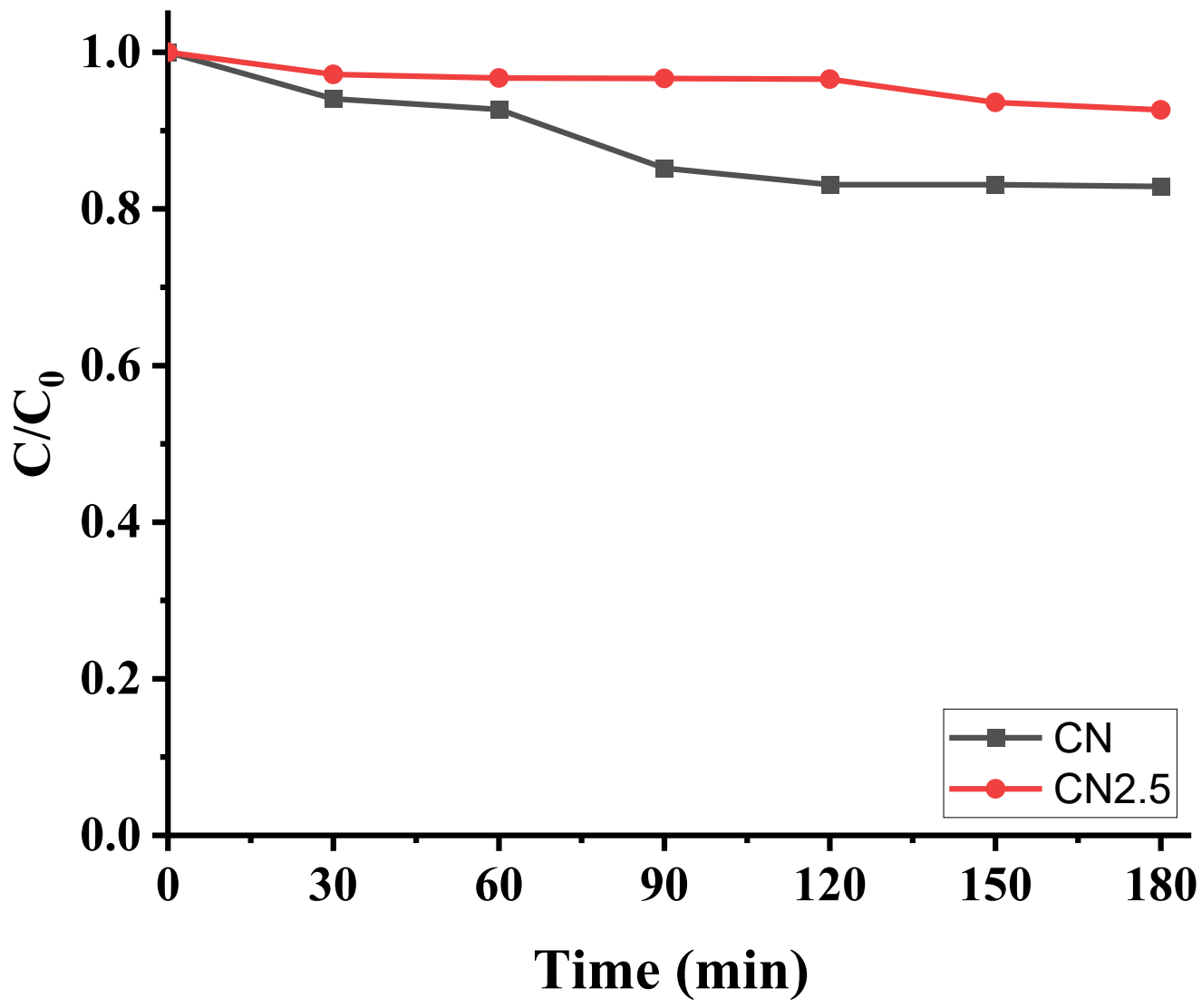

Figure S5. Dark adsorption of DCF on CN and CN2.5.

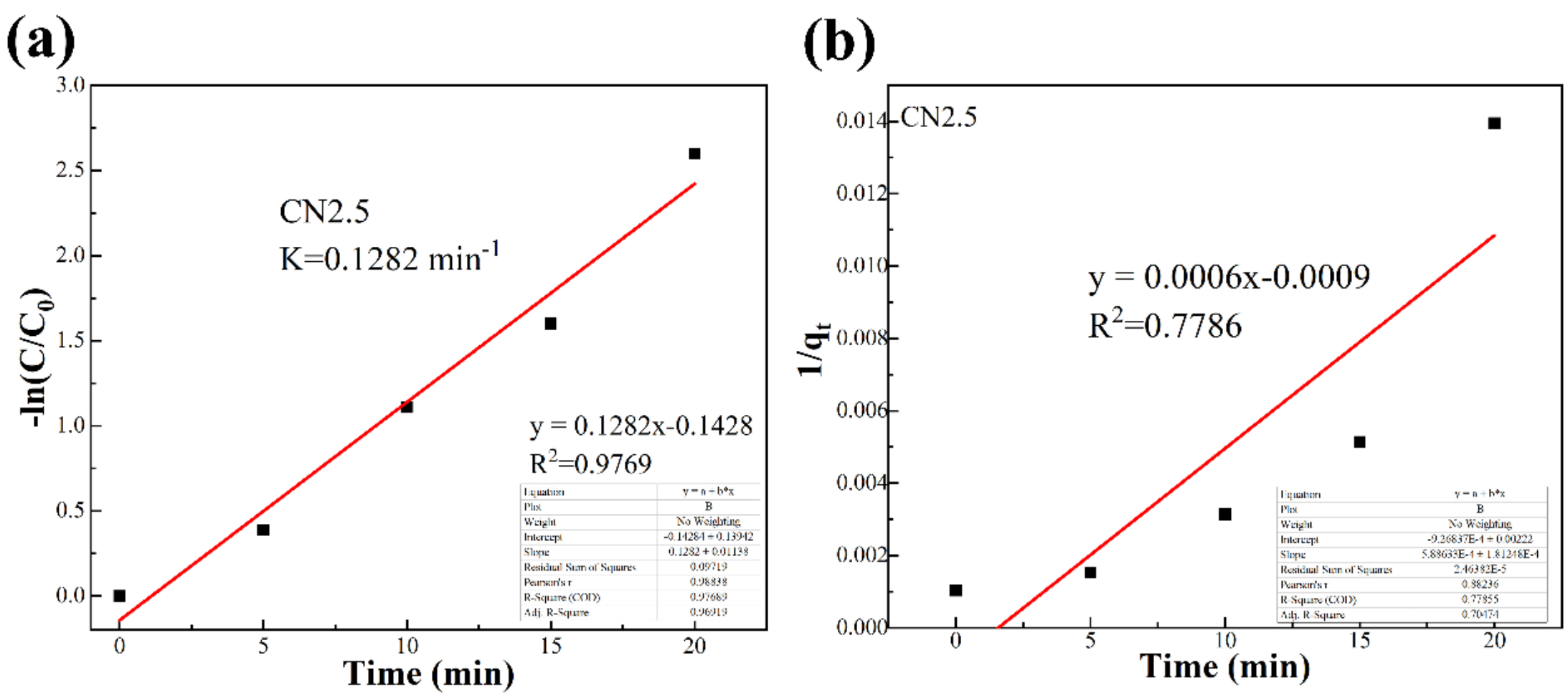

Figure S6. (a)Pseudo-first-order kinetic plot of DCF photodegradation efficiency over CN2.5. (b) Plots of $1/C_t$ versus t based on the pseudo-second-order kinetic model for the degradation of DCF over CN2.5.

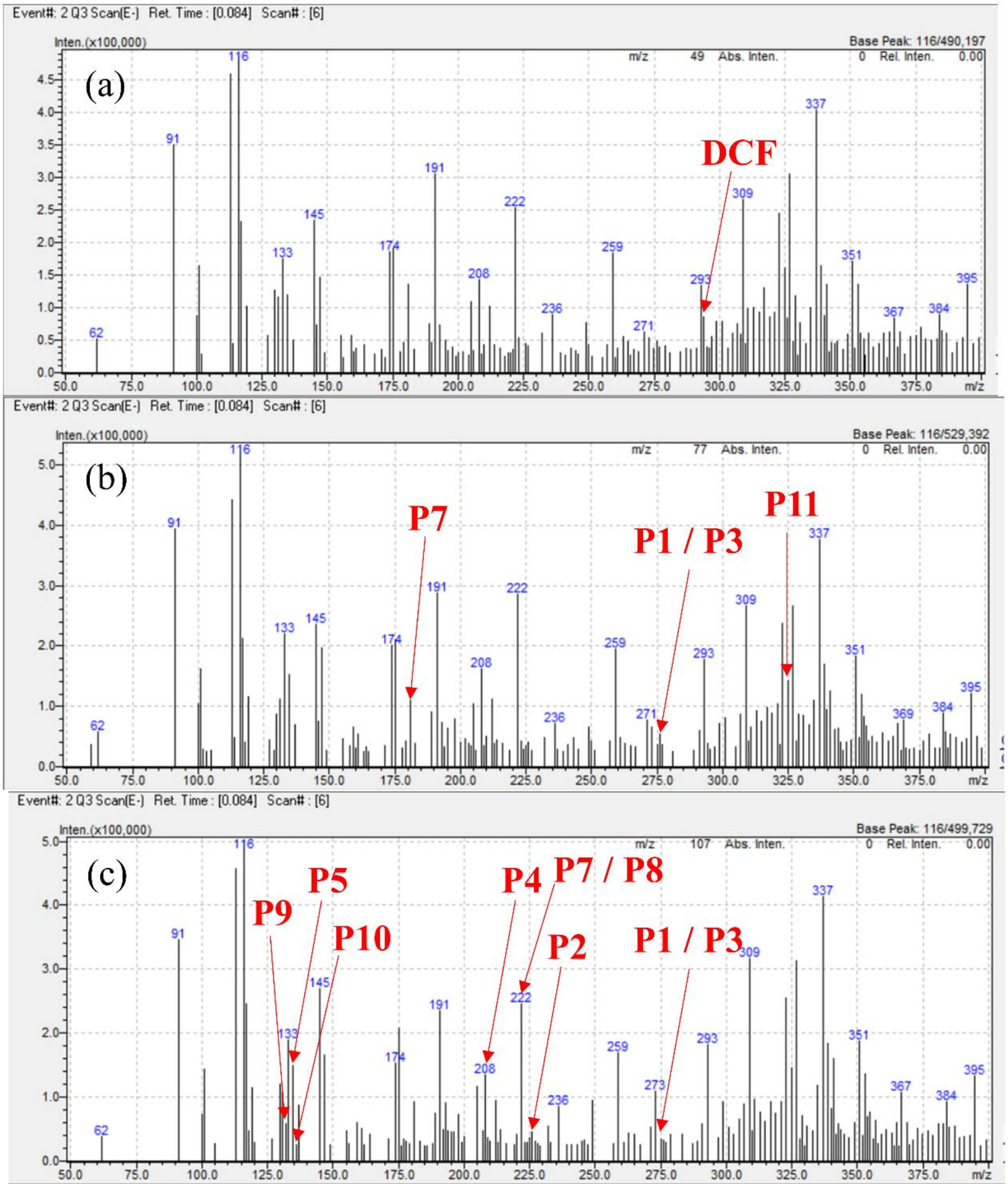

Figure S7. The results of MS of the reaction intermediates of DCF degradation under different degradation times: (a) 0 min (b) 30 min, and (c) 60 min.

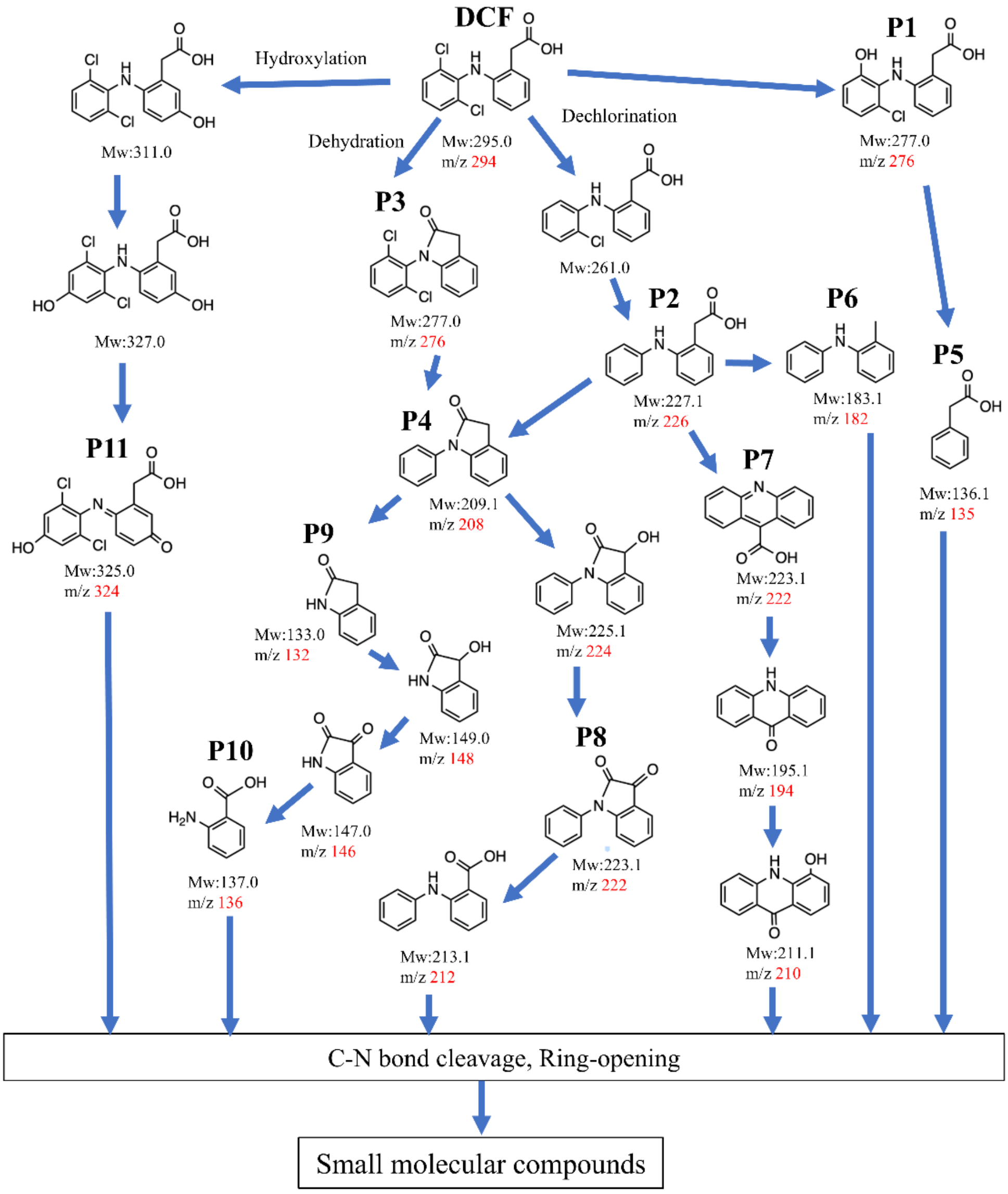

Figure S8. Photocatalytic DCF degradation pathway by mass spectrometry in negative ESI mode.

Table S1. Acute toxicity, bioaccumulation factor, developmental toxicity, and mutagenicity values of DCF and degradation intermediates.

Molecule	Acute Toxicity* LC50 (mg/L)	Bioaccumulation factor	Developmental toxicity**	Mutagenicity***
DCF	2.86	35.14	0.90	0.53
P1	2.13	9.03	0.73	0.50
P2	6.38	16.68	0.85	0.39
P3	10.63	44.41	0.61	0.16
P4	5.06	32.29	0.52	0.41
P5	83.16	1.47	0.48	0.06
P6	0.66	67.70	0.46	0.10
P7	7.30	11.75	0.60	0.76
P8	6.99	20.35	0.48	0.30
P9	58.76	1.99	0.21	0.08
P10	18.93	0.37	0.49	0.15
P11	3.25	15.94	1.02	0.20

*Acute toxicity: LC50 <10 mg/L is Toxic; 10-100 mg/L is Harmful; >100 mg/L is not harmful.[1]

**Developmental toxicity: value >0.5 is developmental toxicant; <0.5 is developmental non-toxicity.

***Mutagenicity: value >0.5 is mutagenicity positive; <0.5 is mutagenicity negative.

Table S2. Comparisons of DCF degradation catalyzed by CN2.5 and previous reported catalysts.

Photocatalysts	Catalyst dosage	[DCF]	Light source	Degradation time	Degradation rate	Ref.
CQDs / g-C_3N_4	10 mg	10 mg/L 50 mL	300 W Xenon lamp	60 min	100%	[2]
Ag / g-C_3N_4	10 mg	100 mg/L 100 mL	300 W Xenon lamp	120 min	100%	[3]
TiO_2	10 mg	9.24 mg/L 50 mL	1500 W Xenon lamp	60 min	100%	[4]
PAA	10 mg	50 mg/L 50 mL	15 W UV lamps	50 min	100%	[5]
gC_3N_4 / NH_2-MIL-125	25 mg	10 mg/L 100 mL	170 $W\cdot M^{-2}$ 384 nm LED	120 min	100%	[6]
TiO_2 / P25	50 mg	25 mg/L 400 mL	175 W UV-A lamp	30 min	80%	[7]
CN2.5	20 mg	6 mg/L 100 mL	6 W 405 nm LED	25 min	99%	This work

Reference

(1) Cai, Z.; Song, Y.; Jin, X.; Wang, C. C.; Ji, H.; Liu, W.; Sun, X. Highly efficient AgBr/h-MoO(3) with charge separation tuning for photocatalytic degradation of trimethoprim: Mechanism insight and toxicity assessment. *Sci Total Environ* **2021**, *781*, 146754. DOI: 10.1016/j.scitotenv.2021.146754.

(2) Liu, W.; Li, Y.; Liu, F.; Jiang, W.; Zhang, D.; Liang, J. Visible-light-driven photocatalytic degradation of diclofenac by carbon quantum dots modified porous g-C3N4: Mechanisms, degradation pathway and DFT calculation. *Water Research* **2019**, *151*, 8-19. DOI: https://doi.org/10.1016/j.watres.2018.11.084.

(3) Zhang, W.; Zhou, L.; Deng, H. Ag modified g-C3N4 composites with enhanced visible-light photocatalytic activity for diclofenac degradation. *Journal of Molecular Catalysis A: Chemical* **2016**, *423*, 270-276. DOI: https://doi.org/10.1016/j.molcata.2016.07.021.

(4) Calza, P.; Sakkas, V. A.; Medana, C.; Baiocchi, C.; Dimou, A.; Pelizzetti, E.; Albanis, T. Photocatalytic degradation study of diclofenac over aqueous TiO2 suspensions. *Applied Catalysis B: Environmental* **2006**, *67* (3), 197-205. DOI: https://doi.org/10.1016/j.apcatb.2006.04.021.

(5) Zhang, L.; Liu, Y.; Fu, Y. Degradation kinetics and mechanism of diclofenac by UV/peracetic acid. *RSC Advances* **2020**, *10* (17), 9907-9916, 10.1039/D0RA00363H. DOI: 10.1039/D0RA00363H.

(6) Muelas-Ramos, V.; Sampaio, M. J.; Silva, C. G.; Bedia, J.; Rodriguez, J. J.; Faria, J. L.; Belver, C. Degradation of diclofenac in water under LED irradiation using combined g-C3N4/NH2-MIL-125 photocatalysts. *Journal of Hazardous Materials* **2021**, *416*, 126199. DOI: https://doi.org/10.1016/j.jhazmat.2021.126199.

(7) Hashim, N.; Natarajan, P.; Ray, A. K. Intrinsic Kinetic Study for Photocatalytic Degradation of Diclofenac under UV and Visible Light. *Industrial & Engineering Chemistry Research* **2014**, *53* (49), 18637-18646. DOI: 10.1021/ie404355k.
